# PD-1^Hi^ CAR-T cells provide superior protection against solid tumors

**DOI:** 10.3389/fimmu.2023.1187850

**Published:** 2023-06-14

**Authors:** Cooper J. Sailer, Yeonsun Hong, Ankit Dahal, Allison T. Ryan, Sana Mir, Scott A. Gerber, Patrick M. Reagan, Minsoo Kim

**Affiliations:** ^1^ Department of Microbiology and Immunology, David H. Smith Center for Vaccine Biology and Immunology, University of Rochester, Rochester, NY, United States; ^2^ Department of Pathology, University of Rochester Medical Center, Rochester, NY, United States; ^3^ Department of Surgery, University of Rochester, Rochester, NY, United States; ^4^ Department of Medicine, Wilmot Cancer Institute, University of Rochester Medical Center, Rochester, NY, United States

**Keywords:** car-t, PD-1, immune checkpoint blockade, T cell migration, cancer immune cell therapy

## Abstract

Chimeric antigen receptor (CAR)-T cell therapy has emerged as a promising treatment option for several hematologic cancers. However, efforts to achieve the same level of therapeutic success in solid tumors have largely failed mainly due to CAR-T cell exhaustion and poor persistence at the tumor site. Although immunosuppression mediated by augmented programmed cell death protein-1 (PD-1) expression has been proposed to cause CAR-T cell hypofunction and limited clinical efficacy, little is known about the underlying mechanisms and immunological consequences of PD-1 expression on CAR-T cells. With flow cytometry analyses and *in vitro* and *in vivo* anti-cancer T cell function assays, we found that both manufactured murine and human CAR-T cell products displayed phenotypic signs of T cell exhaustion and heterogeneous expression levels of PD-1. Unexpectedly, PD-1^high^ CAR-T cells outperformed PD-1^low^ CAR-T cells in multiple T cell functions both *in vitro* and *in vivo*. Despite the achievement of superior persistence at the tumor site *in vivo*, adoptive transfer of PD-1^high^ CAR-T cells alone failed to control tumor growth. Instead, a PD-1 blockade combination therapy significantly delayed tumor progression in mice infused with PD-1^high^ CAR-T cells. Therefore, our data demonstrate that robust T cell activation during the ex vivo CAR-T cell manufacturing process generates a PD-1^high^ CAR-T cell subset with improved persistence and enhanced anti-cancer functions. However, these cells may be vulnerable to the immunosuppressive microenvironment and require combination with PD-1 inhibition to maximize therapeutic functions in solid tumors.

## Introduction

CAR-T cell therapy has become an effective treatment for patients with advanced cancers, including lymphomas, leukemias, and multiple myeloma (1, 2). However, many patients fail to respond in part due to functional exhaustion of the CAR-T cell and failure to persist after infusion (3). Indeed, recent studies have revealed that patients receiving CD19 CAR-T cell products with features of T cell exhaustion, such as co-expression of multiple inhibitory receptors, are not likely to respond to this therapy (4–6). Therefore, CAR-T cell hypofunction driven by inhibitory receptors is likely to be a critical roadblock in the pursuit of translating CAR-T cell therapy into solid tumors, where complete response was achieved in only 4% of patients across available data from current solid tumor CAR-T clinical trials (7).

PD-1 is a cell-surface inhibitory receptor that shares sequence homology with members of the CD28 family of receptors, such as CTLA-4 and ICOS (8). PD-1 is expressed by all T cells during T cell activation and is often associated with T cell dysfunction and exhaustion during settings of chronic infections and cancer (9, 10). Upon ligation to its ligands (PD-L1/2), PD-1 functions by recruiting phosphatases to the immune synapse that dampen activating signals received primarily from the T-cell receptor (TCR) and CD28 activations and their downstream signaling pathways, such as PI3K, RAS, ERK and PLCγ (11, 12). These inhibitory signals from PD-1 negatively affect many aspects of cytotoxic T cell functions, including T cell activation, proliferation, effector cytokine production, T cell metabolism (13–15), migration (16–18), and target cell killing (19–21).

The expression of PD-1 has been implicated in the efficacy of CAR-T cell therapies. Patients with higher proportions of CD8^+^PD-1^+^ T cells in their CAR-T infusion product were less likely to respond to CD19 CAR-T cell therapy against chronic lymphocytic leukemia (4). Elevated proportions of CD8 and CD4 T cells expressing PD-1 and LAG3 found in apheresis starting materials for patients receiving CD19 CAR-T cell therapy were more likely to experience an early treatment failure (6). Interestingly, PD-1 expression level in CD4^+^ CAR-T cells was associated with engraftment and progression-free survival in glioblastoma patients receiving EGFRvIII CAR-T cell therapy (22), suggesting correlations between PD-1 expression and CAR-T cell therapeutic response may be disease type specific.

Several approaches have been tested to relieve the functional impacts of inhibitory receptors on CAR-T cells. However, genetic knockdown of PD-1 expression was shown to impair the proliferation of CAR-T cells *in vitro* (23). Additionally, PD-1 knockout (KO) via CRISPR/Cas9 in TCR-transgenic T cells showed only 5% of T cell survival at 4 months post-infusion, indicating the limited ability of PD-1 KO T cells to persist long-term (24, 25). Instead, multiple clinical trials testing the combination therapy of CAR-T cells with anti-PD-1 blockade are currently underway for solid tumors, including an anti-GD2-CAR for neuroblastoma (NCT01822652), anti-mesothelin CAR for malignant pleural diseases (NCT02414269) and a HER2-tareted CAR for sarcoma (NCT04995003) (26–31).

The goal of this study is to better understand the underlying mechanisms for PD-1-mediated alterations in CAR-T cell functions. We addressed critical knowledge gaps regarding the biological consequences of PD-1 expression in therapeutic T cells that determine the function and fate of PD-1^+^ CAR-T cells in solid tumors. We observed a heterogeneous expression of PD-1 (PD-1^high^ vs. PD-1^low^) in *in vitro* activated CAR-T cells. PD-1^high^ CAR-T exhibited greater cytotoxic effector functions and superior tumor retention after adoptive transfer. Importantly, the increased cancer control of PD-1^high^ CAR-T cells *in vivo* was observed only when anti-PD-1 blocking antibody was co-injected with PD-1^high^ CAR-T cell infusion. Thus, our data suggest that augmented CAR-T cell functions in solid tumors may paradoxically depend on a unique subpopulation of PD-1^high^ CAR-T cells that possess optimal anti-cancer functions and that a combination strategy with a PD-1 inhibition is required to maximize therapeutic functions of CAR-T cells.

## Method

### Antibodies and reagents

UltraComp eBeads, CellTrace CFSE, CellTrace Violet, CellTrace FarRed and AF700-CD101 (Moushi101) were purchased from Invitrogen. Recombinant Human ICAM-1/CD54 Fc Chimera Protein, Recombinant Mouse CXCL12/SDF-1 alpha Protein, Recombinant Mouse PD-L1/B7-H1 Fc Chimera Protein, Recombinant mouse CXCL10/IP-10/CRG-2, and Recombinant Human ErbB2/Her2 Fc Chimera Protein were purchased from R&D Biosystems. anti-mouse PD-1 (29F.1A12), rat IgG2a isotype control (2A3), anti-mouse IFNy (XMG1.2) and anti-mouse LFA-1 (M17/4) were purchased from Bioxcell. Monensin 1000x solution and Brefeldin A 1000x solution were purchased from eBiosciences. The antibody BV421-PD-1 (EH12.1) was purchased from BD Biosciences. Antibodies against FITC-CD223 (C9B7W), AF488-CD25 (PC61.5), FITC-Perforin (eBioOMAK-D), and PE-anti-Human IgG Fc secondary antibody were purchased from eBioscience. Antibodies against APC-Annexin-V, PE-Cy7-CD69 (H1.2F3), PE-Cy7-CD107a (1D4B), and purified NA/LE Hamster anti-mouse CD3e (145-2C11) was purchased from BD Pharmingen. Antibodies against BV421-PD-1 (RMP1-30), AF647-CD340 (24D2), APC-CD45.1 (A20), APC-CD366 (RMT3-23), purified anti-mouse CD28 (37.51), BV711-CD274 (10F.9G2), AF647-CD54 (YN1/1.74), BV605-IFNγ (XMG1.2), PE-TNFα (MP6-XT22), PE-Ly108 (330-AJ), APC-CD44 (IM7), PE-CD11a/CD18 (LFA-1, H155-78), APC-CD184 (1.276F12), APC-Granzyme B (QA16A02), FITC-CD366 (F38-2E2), 7-AAD Viability Staining, PE-Cy7-CD62L (MEL-14), AF647-CCR7 (4B12), BV711-CX3CR1 (SA011F11), BV421-CXCR3 (CXCR3-173), BV421-CXCR5 (L138D7), BV605-CD127 (A7R34), APC-PD-1 (RMP1-30), BV711-PD-L1 (10F-9G2), APC-IL-2 (JES6-5H4), APC-Ki67 (16A8), APC-Cy7-CD45 (30-F11) and Recombinant Mouse IFNγ was purchased from BioLegend. RetroNectin was purchased from Takara. IL-2 was purchased from PeproTech.

### Mice

B6.SJL-*Ptprc^a^ Pepc^b^*/BoyJ (B6-CD45.1) and C57BL/6-Tg(UBC-GFP)30Scha/J (B6-GFP) mice were purchased from the Jackson Laboratory. Human-HER2 transgenic mice (hHER2-TG) were obtained from Genentech (32). Genotyping for each strain was performed according to the corresponding reference. All mice were further backcrossed with C57BL/6J mice for at least more than 12 generations. Mice were maintained in a pathogen-free environment of the University of Rochester animal facility, and the animal experiments were approved by the University Committee on Animal Resources at the University of Rochester (Rochester, NY).

### Cell culture

B16-hHER2 cells were cultured in DMEM supplemented with 10% FBS, 1% penicillin/streptomycin and 1 mg/mL G418 (Gibco). BT474 cells were cultured in DMEM supplemented with 10% FBS and 1% penicillin/streptomycin. E0771-hHER2 cells were cultured in RPMI 1640 supplemented with 10% FBS, 1% antibiotic-antimycotic (Gibco), 2 mM L-glutamine (Gibco), 20 mM HEPES buffer (Gibco), 1% MEM Non-Essential Amino Acids (Gibco), 50 µM ß-mercaptoethanol (Sigma-Aldrich) and 1 mg/mL G418 (Gibco). Platinum-E cells were cultured in DMEM supplemented with 10% FBS, 1% penicillin/streptomycin, 20 mM HEPES buffer (Gibco), 1% MEM Non-Essential Amino Acids (Gibco) and 50 µM ß-mercaptoethanol (Sigma-Aldrich)1 μg/ml of puromycin and 10 μg/ml of blasticidin S HCl. Purified mouse and human CD8^+^ T cells were cultured in RPMI 1640 supplemented with 10% FBS, 1% antibiotic-antimycotic (Gibco), 2 mM L-glutamine (Gibco), 20 mM HEPES buffer (Gibco), 1% MEM Non-Essential Amino Acids (Gibco), 50 µM ß-mercaptoethanol (Sigma-Aldrich) and 80 U/mL IL-2. All cells were maintained at 37°C in 5% CO_2_. For E0771 and B16-hHER2 stable cell line, B16F10 mouse melanoma cells (ATCC) were transfected with human HER2 pcDNA3.1 mammalian expression plasmid (Addgene #16257) using Lipofectamine 2000 according to the manufacturer’s protocol. Antibiotic selection (G418, 1 mg/mL) was added to the media 24 hours post-transfection. Cells were stained with anti-human HER2 antibody and sorted for the top 5% of HER2 expressers. Sorted cells were plated in 96-well plates at 1 cell per well to generate single cell clones. When thawed, stable cells were cultured in the presence of G418, and used within 2 weeks.

### T cell purification and activation

Mouse CD8 T cells were purified from single-cell suspensions of the spleen and lymph nodes of C57BL/6 mice. Single-cell suspensions were prepared by mechanical disruption in a 70μm cell strainer. CD8 T cells were enriched by magnetic-bead depletion with rat anti-mouse MHC II antibody (M5/114) and rat anti-mouse CD4 antibody (GK1.5), followed by sheep anti-rat IgG magnetic beads (Invitrogen 11035). Isolated CD8 enriched T cells were cultured in complete RPMI medium supplemented with 80 U/mL IL-2 and activated with plate-bound CD3e Ab (1 μg/mL) and CD28 Ab (1.6 μg/mL) for 24 hours.

### CAR-T cell transduction

Activated mouse CD8^+^ T cells were transduced with a 3^rd^ generation trastuzumab (4D5; anti-HER2)-based CAR with CD8 transmembrane domain, CD28 and 4-1BB costimulatory domains, and CD3ζ (33). CAR sequence was first cloned into the pMSCV vector (631461, Clontech). Retrovirus was generated using the Platinum-E packaging cell line. Platinum-E cells were plated at 0.8x10^6^ cells per well in a 6-well plate. The following day, Platinum-E cells were transfected with HER2-CAR pMSCV using Lipofectamine 2000 according to the manufacturer’s protocol. Media was swapped 6 hours post-transfection. Filtered (0.45 μM) Retroviral-containing supernatants were collected 24- and 48-hours post-transfection. For T cell transduction, 1x10^6^ activated CD8 T cells were plated on RetroNectin (Takara) coated 12-well plates (10 μg/mL) with retrovirus-containing supernatants, supplemented with 80 U/mL IL-2, and spun twice at both 24- and 48 hours post-transfection at 2500 RPM. T cells are then detached from RetroNectin at least 24 hours post transduction and analyzed for HER2-CAR expression. After transduction, CAR-T cell were cultured and expanded with 80 U/mL IL-2 and plate-bound CD3e Ab (1 μg/mL). Cells were collected, dead cells removed, washed and plated on new CD3-coated plates with fresh media every 2-3 days.

### CAR-T cell patient samples

Peripheral blood mononuclear cells (PBMCs) and plasma samples are being collected from patients with B-cell lymphoma that received axicabtagene ciloleucel (2x10^6^ cells/kg). Patients consent for blood collection under the University of Rochester Medical Center Wilmot Cancer Center’s lymphoma research program tissue collection protocol ULAB-03012 in accordance with the Institutional Review Board. Peripheral blood was obtained in sodium heparin tubes before treatment and after axicabtagene ciloleucel infusion from forty patients 5 days prior to CAR-T cell infusion. T cells were isolated from the blood using STEMCELL Human T cell isolation kit per manufacturers protocol. Cells were cryopreserved for later analysis. These same patients were undergoing CAR-T cell therapy treatment. CAR-T cells are prepared commercially and returned to the Wilmot Cancer Center for infusion. After infusion, some cells typically remain in the infusion tubing and storage bag. These cells are usually discarded but were recovered and used in the current studies. After completion of CAR-T cell infusion, the infusion bag was washed with PBS to obtain residual cells and cryopreserved for later analysis for inhibitory receptor expression.

### Flow cytometry and cell sorting

To sort PD-1^high^ and PD-1^low^ CAR-T cells, CAR-T cells were collected from cell culture and washed in PBS. To detect CAR expression level on T cells, 1x10^6^ CAR-T cells were pre-incubated with 2 μg Fc blocker (BD Biosciences) for 10 min at 4°C and incubated with recombinant human ErbB2/Her2 Fc chimera protein (R&D systems) at 4°C for 30 min. After washing in PBS, the cells were incubated with PE-conjugated anti-human IgG Fc secondary antibody (eBioscience) and BV421-conjugated mouse anti-PD-1 (Biolegend) at 4°C for 30 min. Cells were washed and sorted by BD FACSAria II cell for CAR-positive cells and the top and bottom 20% of PD-1 levels. For intracellular cytokine staining, cancer cells were labeled with CFSE per manufacturers protocol and plated and cultured overnight. CAR-T cells were sorted by their PD-1 expression and added to the plate for 4 hours. CAR T cells were added to anti-CD3/CD28 coated plates for intracellular IL-2 experiments. Cells were cultured in the presence of monensin (1:1000) and brefeldinA (1:1000). Intracellular proteins or cytokines were stained with following antibodies at RT for 1 hr after fixation and permeabilization via eBioscience Intracellular Fixation and Permeabilization Buffer Set: IFN-γ-BV421 (XMG1.2), TNF-α-BV421 (MP6-XT22), FITC-Perforin (eBioOMAK-D), APC-IL-2 (JES6-5H4), APC-Ki67 (16A8) and GranzymeB-APC (QA18A28). Mean fluorescence intensity or percent of cells expression cytokines was determined by flow cytometry. For analytical cytometry, stained cells were detected by BD LSR II or Symphony A1 flow cytometer and analyzed via FlowJo.

### *In Vitro* T cell migration imaging

Cell migration chambers (Millicell EZ slide eight-well glass, Millipore) were prepared by coating with Protein A (Invitrogen, 20 μg/mL), ICAM-1 (2.75 μg/mL), CXCL10 and CXCL12 (400 ng/mL) in PBS. 5x10^4^ PD1-sorted CD8^+^ T cells were plated in Leibovitz’s medium supplemented with 2 mg/mL D-glucose in a 37°C chamber. Video microscopy was conducted using a TE2000-U microscope (Nikon) coupled to a CoolSNAP HQ CCD camera with a 10x objective and 0.45 numerical aperture. T Cells and cancer cells were stained with CellTrace CFSE, CellTrace Violet, or CellTrace FarRed per manufacturers protocol (ThermoFisher). T cells were plated in L-15 medium at least 20 min at 37°C before imaging. For T cells alone, bright field or DIC images were acquired every 15 sec for 20 min. For T cell and cancer cell coculture movies, the stained cancer cells were plated 24 hours before the start of imaging. Bright field and fluorescent images were captured every 30 seconds for at least 2 hours.

### *In Vitro* T cell migration analysis

Migration analysis was performed in Volocity software (PerkinElmer). In order to select which T cell tracks to analyze in Volocity, we excluded cells that are smaller than 10 μm and greater than 200 μm. Additionally, static cells were ignored, broken tracks were automatically joined, and cell tracks less than 20 μm were excluded. We also omitted T cells that were migrating for less than 5 minutes of the 20-minute movie and removed cells that Volocity incorrectly tracked. For PD1 hi and lo T cell and cancer cell coculture movies, cells to analyze were selected using their fluorescence (CellTrace Violet or CellTrace FarRed). The velocity (μm min^-1^), displacement (net displacement, μm), track length (total path length, μm), and meandering index (net displacement/track length) of each cell track was measured.

### *In Vitro* 3D tumoroid assay

Tumor spheroids were generated using AggreWell 800 culture plates (STEMCELL). Wells were pre-treated with anti-adherence rinsing solution (STEMCELL) and centrifuged at 1200 RPM for 5 minutes. Wells were rinsed with media, and BT474 breast cancer cells were added to generate spheroids with between 4,000 and 5,000 cells per microwell, and spun at 1200 RPM for 5 minutes. After 24 hours, the spheroids were collected by gentle pipetting and allowed to settle in a 1.4 mL Eppendorf tube and excess media was aspirated. HER2-CAR T cells were stained with CellTrace FarRed or CellTrace Violet (ThermoFisher) per manufacturers protocol. Stained CAR-T cells were resuspended in 50 uL of complete RPMI and mixed with the tumor spheroids. 50 ul of Matrigel (Corning) was added to the stained CAR-T cell/spheroid mixture and mixed thoroughly. The total volume (100 μL) pipetted to the center of a ΔT-dish (Bioptechs), and incubated at 37°C and 5% CO_2_ for 30 minutes to allow the gel to polymerize into a 3D matrix. Once solidified, 1 mL of Leibovitz’s media supplemented with 20 mg/mL glucose was added to the dish and live-imaging time-lapse microscopy was performed. Video microscopy was conducted using a TE2000-U microscope (Nikon) coupled to a CoolSNAP HQ CCD camera with a 10x objective and 0.45 numerical aperture. The ΔT-dish was maintained in a 37°C chamber. Brightfield and fluorescent images were acquired every 60 seconds for up to 24 hours. For quantification, a single region of interest (ROI) was drawn around the tumoroid, and the mean fluorescence intensity increase within the ROI over time for PD-1^high^ (green) and PD-1^low^ (red) was measured.

### Killing assay

Cancer cells were labeled with CFSE per manufacturers protocol. CAR-T cells were sorted on PD-1 expression. The cells were added to 24-well TC-treated plates and left overnight at various effector to target ratios. Anti-PD-1 and anti-IFNγ antibodies were added at a final concentration of 20 μg/mL in specified experiments. All cells were recovered from the plate after trypsinization into separate conical tubes. Cells were washed in PBS and 7-AAD was added to flow tubes. Cancer cell death (CFSE^+^7-AAD^+^) was measured by flow cytometry. Specific cell death was calculated using the following formula: (CAR-T Target % - Control Target %)/(100 – Control Target %) x 100

### Degranulation assay

Cancer cells were labeled with CFSE per manufacturers protocol. CAR-T cells were sorted by PD-1 expression and added to the plate. Cells were cultured for 4 hours in the presence of monensin (1:1000) and PECy7-conjugated anti-CD107a (1 μL per mL of media) added directly into the media. T cells were recovered from the plate, washed, and CD107a expression was detected by flow cytometry.

### Conjugation assay

Cancer cells were labeled with CFSE per manufacturers protocol. Sorted CAR-T cells were stained with CTFR. Labeled cancer cells and CAR-T cells were mixed in an Eppendorf tube containing 100 μL total volume for 2 hours. Cells were fixed directly in the Eppendorf tube by addition of 4% PFA (Paraformaldehyde, 16% w/v aq. Soln., methanol free, ThermoFisher Scientific) for 30 min. Cells were gently spun and washed and transferred to a flow tube. Events double positive for CTFR and CFSE were identified to determine the percent of CAR-T cells in conjugation with cancer cells.

### *In vivo* assay

HER2-TG mice were inoculated with 5x10^5^ B16-hHER2. After 7 days, approximately 1 million CAR T cells were injected either intravenously via retro-orbital injection or intratumorally in 10 μL PBS. For tumor growth analysis, tumors were measured using electronic calipers at the time of T cell injection and every 2 days thereafter. Tumor were allowed to grow for 7 or 8 more days or until tumors reached their endpoints. Mice were treated with 100 μg of anti-PD-1 antibody (29F.1A12) or rat IgG2a isotype control (2A3) at tumor day 7 and 11 by I.P. injection. Tumor volumes were estimated using the following calculation: (l x w^2^ x 0.52). At experimental endpoints, mice were sacrificed and tumors were removed, manually disrupted, and digested in collagense/dispase (Roche) for 30 min at 37°C. Digested tumor suspensions were filtered through a 100 μm strainer, washed, and analyzed using flow cytometry.

### Statistical analysis

All statistical tests were performed with GraphPad Prism (v9). Statistical analysis was performed using Two-way ANOVA, ordinary One-Way ANOVA with a Tukey’s multiple-comparison post-test, unpaired *t*-test, and Mann– Whitney test when appropriate. Differences were considered significant when *P* values were <0.05.

## Results

### CAR-T cell production induces heterogenous PD-1 expression

**Figure 1:**
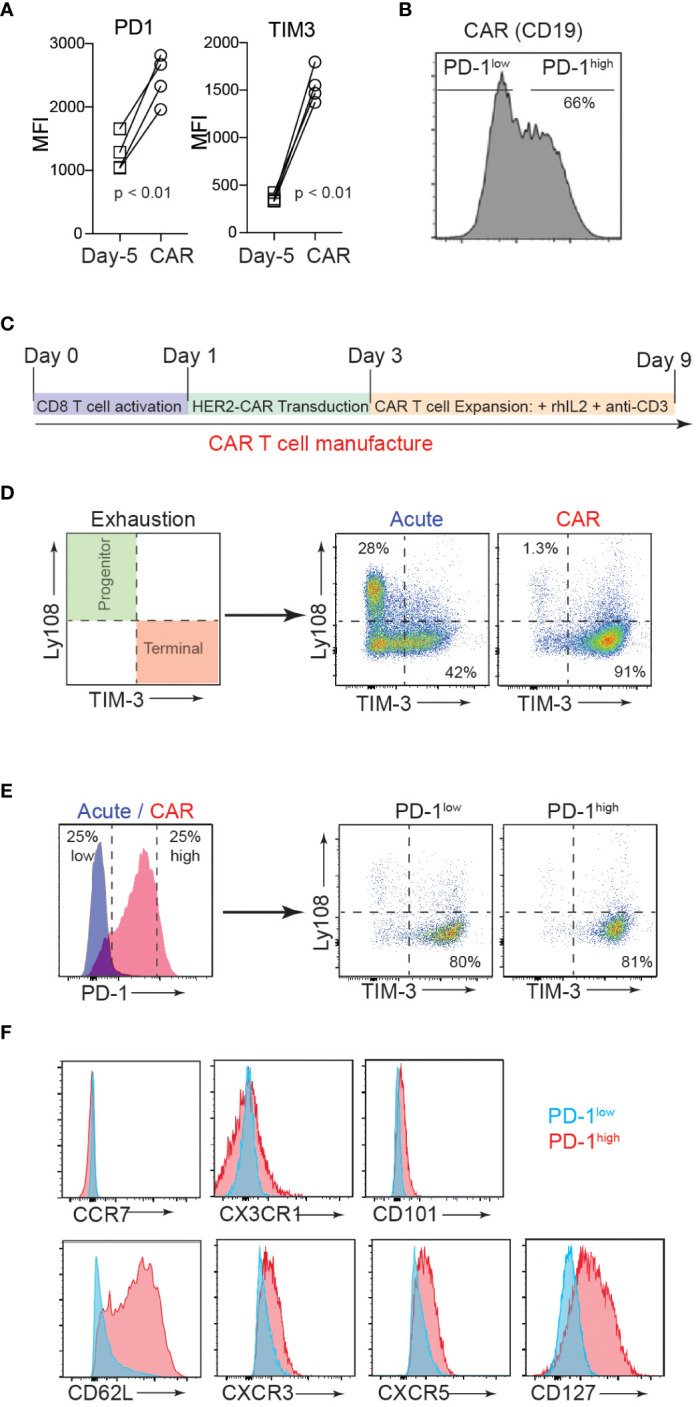
CAR-T cell production induces heterogenous PD-1 expression. **(A)** T cells were isolated from axicabtagene ciloleucel patient peripheral blood 5 days prior to infusion (D-5, squares) and the CAR-T cell infusion bag (CAR, circles). Flow cytometry analysis of surface inhibitory receptors PD-1 and TIM3 mean fluorescence intensity (MFI) on four patient-derived CD19 CAR T cell samples is depicted. *P* value was determined by paired, two-sided Student’s *t*-test. **(B)** Representative flow cytometry histogram of PD-1 expression in CD19 CAR T cell infusion product. **(C)** Schematic of murine CD8 CAR-T cell manufacturing process. **(D)** Flow cytometry analysis showing the surface expression of progenitor (Ly108^+^TIM3^-^) and terminal (Ly108^-^TIM3^+^) exhausted subsets after murine CAR-T cell manufacturing (CAR) or expansion in the absence of plate-bound anti-CD3 (Acute). **(E)** Flow cytometry analysis showing surface PD-1 expression after murine CAR-T cell manufacturing (*left*) and exhaustion subsets of high and low PD-1 expressors (*right*). **(F)** Representative flow cytometry histograms showing expression of select surface markers for PD-1^low^ (blue) and PD-1^high^ (red) CAR T cells.

Exhausted T cells express PD-1, which mainly limits immunopathology in the setting of chronic TCR stimulation (34). Similarly, given the repeated TCR activations and continued cytokine stimulation involved, *ex vivo* manufacturing procedures for CAR-T cells often inevitably induce terminal differentiation and senescence of T cells with cell surface expression of exhaustion markers (35). Despite the clear role of PD-1/PD-L1 in regulating the anti-tumor responses of effector T cells, the biological impact of this inhibitory receptor expression on CAR-T cell function remains largely unexplored. To investigate how PD-1 controls the function of CAR-T cells, we first measured PD-1 expression on naïve T cells isolated from CD19 CAR-T cell (axicabtagene ciloleucel) patients (on day -5 before CAR-T cell injection) and compared the PD-1 expression level with that of their CAR-T cell product. Flow cytometry analysis confirmed a significantly higher (**Figure 1A**), and heterogeneous expression pattern for PD-1 (PD-1^high^ vs. PD-1^low^) in the CAR-T cell infusion products compared to the corresponding naïve T cells (**Figure 1B**).

The current therapeutic T cell manufacturing process (both for tumor-infiltrating lymphocyte (TIL) and CAR-T cell therapy) includes the selection and activation of T cells from patient apheresis products using CD3/CD28 beads and large clinical scale T cell expansion with high concentrations of IL-2 (36). We adapted this process to generate trastuzumab (4D5; anti-human HER2)-based murine hHER2-CAR-T cells (**Supplementary Figure 1A**) (33). *In vitro* activated mouse CD8 T cells were retrovirally transduced with the CAR at 90% efficiency (**Supplementary Figure 1B**). Coincubation of E0771 mouse breast cancer cell line, which stably expresses human HER2 antigen (E0771-hHER2), with hHER2-CAR expressing mouse CD8 T cells induced dramatic cytotoxicity compared to the control CD8 T cells (**Supplementary Figures 1C-E**), confirming that HER2-CAR expression redirects CD8 T cells to eliminate HER2-expressing target cells. After transduction, HER2-CAR T cells were then expanded *in vitro* with anti-CD3 and recombinant IL-2 to replicate the activation and expansion method used in relevant clinical trials (**Figure 1C**) (36). With this *in vitro* T cell activation protocol, we found that the mouse CAR-T cell product expressed cell surface markers that traditionally represent a “terminally exhausted” T cell population (TIM3^+^Ly108^-^), while “acute” *in vitro* activation of T cells (CAR-T cell expansion in the absence of persistent anti-CD3-mediated TCR activation (37, 38)) induced a “progenitor exhausted” phenotype (**Figure 1D**) (39). Consistent with the data for human CAR-T cells, mouse CAR-T cells expressed heterogeneous levels of surface PD-1 (PD-1^high^ vs. PD-1^low^) and both PD-1^high^ and PD-1^low^ CAR T cells showed a predominantly “terminally exhausted” phenotype and (**Figure 1E**). Flow cytometry analyses of CAR-T cells further revealed that compared to PD-1^low^ CAR T cells, PD-1^high^ CAR T cells display elevated levels of a lymph node homing adhesion molecule, CD62L, but similar expression levels of CCR7, CX3CR1, and CD101 (**Figure 1F**). PD-1^high^ CAR T cells also expressed slightly elevated levels of CXCR3, CXCR5, and CD127 (**Figure 1F**). Collectively, these results indicate that both human and mouse CAR T manufacturing promotes phenotypic signs of exhaustion, yet a spectrum of PD-1 expression and other important surface markers, that are potentially associated with distinct functionalities.

### PD-1^high^ CAR-T cells show enhanced engagement with target cells and superior cytotoxicity

**Figure 2:**
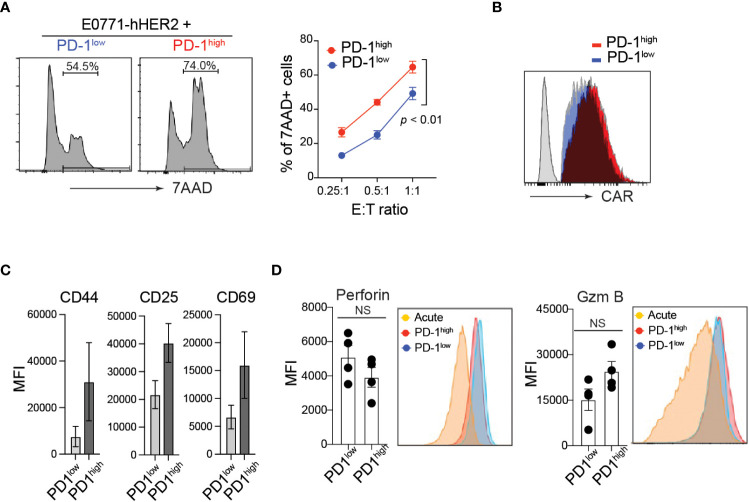
Elevated cytotoxicity of PD-1^high^ CAR-T cells. **(A)** Representative flow cytometry analysis of CFSE-labeled hHER2-E0771 positive for 7-AAD staining after 16-hour co-culture with sorted PD-1^high^ or PD-1^low^ CAR-T cells (*left*) and quantification at stated effector to target ratios (*right*) from three independent experiments in triplicate is shown (mean +/- SEM). *P* value was determined by one-way ANOVA. **(B)** Representative flow cytometry data of surface hHER2-CAR expression on non-transduced T cells (gray) and PD-1^low^ (blue)- or PD-1^high^ (red)-CAR-T cells. **(C)** Flow cytometry MFI of CD44, CD25, and CD69 cell surface expression on PD-1^high^ and PD-1^low^ CAR-T cells from four independent experiments is depicted (mean +/- SEM). **(D)** Representative flow cytometry analysis of intracellular levels of perforin and Granzyme B in PD-1^high^ and PD-1^low^ CAR-T cells and MFI from 4 independent experiments is shown (mean +/- SEM). NS; not significant. *P* value was determined by unpaired, Student’s *t*-test.

PD-1 expression is often associated with functional exhaustion of *in vitro* activated T cells chronically stimulated with high levels of plate-bound anti-CD3 (37, 38). To test this in our *in vitro* manufactured CAR-T cells, CD8 T cells were transduced with anti-HER2 CAR and sorted based on their cell surface PD-1 expression level (**Supplementary Figure 1F**). Unlike our prediction, PD-1^high^ hHER2-CAR-T cells exhibited superior cytotoxicity against E0771-hHER2 cells (**Figure 2A**). Note that PD-1^high^ and PD-1^low^ hHER2-CAR-T cells used in the cancer-killing assay expressed comparable cell surface levels of CAR (**Figure 2B**).

Although PD-1 expression is often used as an indication of T cell exhaustion, it is quickly upregulated on the cell surface within 24 h of T cell activation. Thus, PD-1 can also serve as a marker of T cell activation both *in vitro* and *in vivo* (40). Indeed, PD-1^high^ hHER2-CAR-T cells showed enhanced T cell activation compared with PD-1^low^ hHER2-CAR-T cells (**Figure 2C**). To test whether the enhanced cytotoxicity of PD-1^high^ CAR-T cells is associated with the differential expression of anti-cancer effector molecules after T cell activation, we measured intracellular perforin and granzyme B levels. Flow cytometry analysis of intracellular molecules revealed that PD-1^high^ hHER2-CAR-T cells and PD-1^low^ hHER2-CAR-T cells express comparable levels of effector molecules (**Figure 2D**).

**Figure 3:**
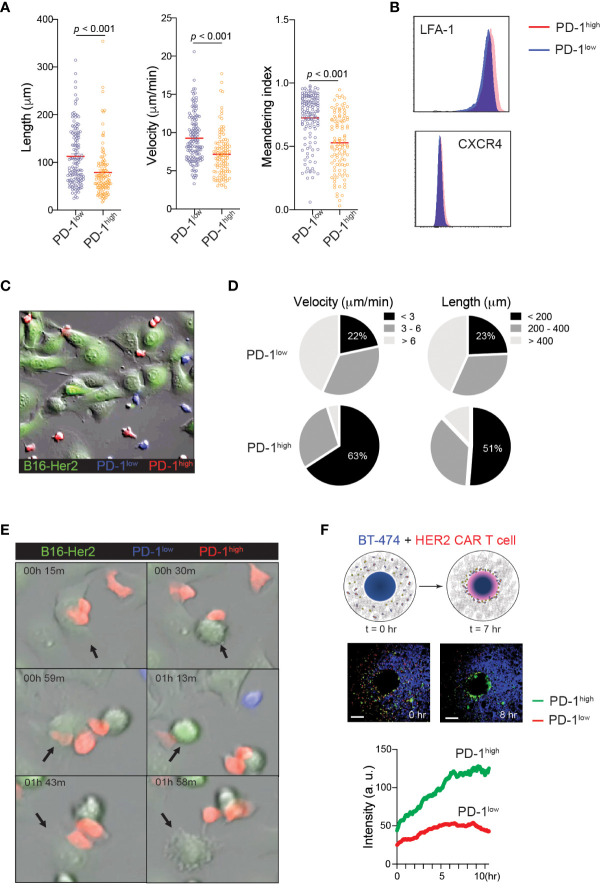
Distinct motility patterns of PD-1^high^ vs. PD-1^low^ CAR-T cells. **(A)** Track length, velocity, and meandering index were calculated from PD-1^high^ and PD-1^low^ CAR-T cells migration on ICAM-1 and CXCL12 coated dishes. Data shown are representative results from one of 4 independent experiments. Solid red line indicates the mean, and *P* value was determined by unpaired, two-sided Student’s *t*-test. **(B)** Representative flow cytometry data showing surface expression of LFA-1 and CXCR4 on PD-1^high^ and PD-1^low^ CAR-T cells. **(C)** Time-lapse sequencing of PD-1^high^ (red) and PD-1^low^ (blue) CAR-T cells with hHER2-expressing B16F10 cells (green) was imaged every 30 seconds for 2 hours. **(D)** Velocity (*left*) and track length (*right*) for PD-1^high^ and PD-1^low^ CAR-T cells were quantified. Cell tracks for PD-1^high^ and PD-1^low^ CAR-T cells were stratified into slowest (<3 μm/min), middle (3-6 μm/min), and fastest (> 6 μm/min) migrating cells and shortest (<200 μm), middle (200-400 μm) and furthest (> 400 μm) migrators while in the presence of target cancer cells. **(E)** Selected still images from the cell migration movie 2 indicating cytotoxic events of PD-1^high^ and PD-1^low^ CAR-T cells targeting hHER2-B16F10 cancer cells on ICAM-1 and CXCL12 coated plates. **(F)** Schematic of *in vitro* 3D CAR-T cell migration assay. hHER2-CAR-T cells swarm to cognate tumoroids in Matrigel over time *(upper)*. Still images from the movie showing the start point (0 hr) and 8 hours later showing increased engagement of PD-1^high^ (green) CAR-T cells with BT474 tumoroids (*middle)*. A single region of interest was drawn around the tumoroid, and mean fluorescence intensity was quantified for recruited PD-1^high^ (green) and PD-1^low^ (red) CAR-T cells over time *(lower).* Data represent 4 experiments with BT474 and HER2-B16 (mouse) tumoroids with similar results.

The finding that PD-1^high^ CAR-T cells exert stronger tumoricidal functions than PD-1^low^ CAR-T cells despite the similar cell surface levels of CAR and intracellular levels of anti-cancer effector molecules suggests the presence of unique T cell-target cell interaction patterns that may promote T cell-mediated killing in PD-1^high^ CAR-T cells. Indeed, elevated PD-1 expression has been previously implicated in the altered migration patterns of cytotoxic T cells in viral infection and cancer (17, 41). To investigate the impact of PD-1 expression on CAR-T cell motility, PD-1^high^ and PD-1^low^ hHER2-CAR-T cells were allowed to migrate on ICAM-1 + CXCL12 coated cover glass. PD-1^high^ hHER2-CAR-T cells showed decreases in track length, cell velocity, and cell meandering index compared to PD-1^low^ hHER2-CAR-T cells (**Figure 3A** and **Movie 1**). The reduced cell motility patterns were unlikely to be due to differential expression levels of the chemokine receptor CXCR4 or integrin LFA-1 (**Figure 3B**). The IFNγ-CXCL10-CXCR3 axis has been implicated in the recruitment of T cells to sites of inflammation and responses to immunotherapy (42, 43). Indeed, PD-1^high^ CAR T cells express slightly elevated levels of chemokine receptor CXCR3 (**Figure 1F**), which may be associated with improved recruitment and retention to tumor sites. However, like the experiments with CXCL12, *in vitro* T cell motility assay with ICAM-1 and CXCL10 coated cover glass showed shorter track length, lower cell meandering index, and slower velocity in PD-1^high^ CAR T cells (**Supplementary Figure 2**). Therefore, our data suggest that the difference in motility is cell intrinsic and not dependent on their response to differential chemokine signals. Interestingly, the reduced velocity of PD-1^high^ CAR-T cells concurred with more stable cell-cell interactions and a prolonged dwelling time within close proximity to target tumor cells (**Figures 3C-E** and **Movie 2**). Furthermore, the unique migration pattern of PD-1^high^ CAR-T cells led to rapid and greater T cell accumulation toward the HER2^+^ BT474 spheroids in 3D migration assays (**Figure 3F** and **Movie 3**). Therefore, our data suggest that CAR-T cells undergo significant molecular and cellular reprogramming during the *in vitro* manufacturing process and that the intrinsic changes in the motility of PD-1^high^ subpopulations allows for more stable interaction with target cells.

**Figure 4:**
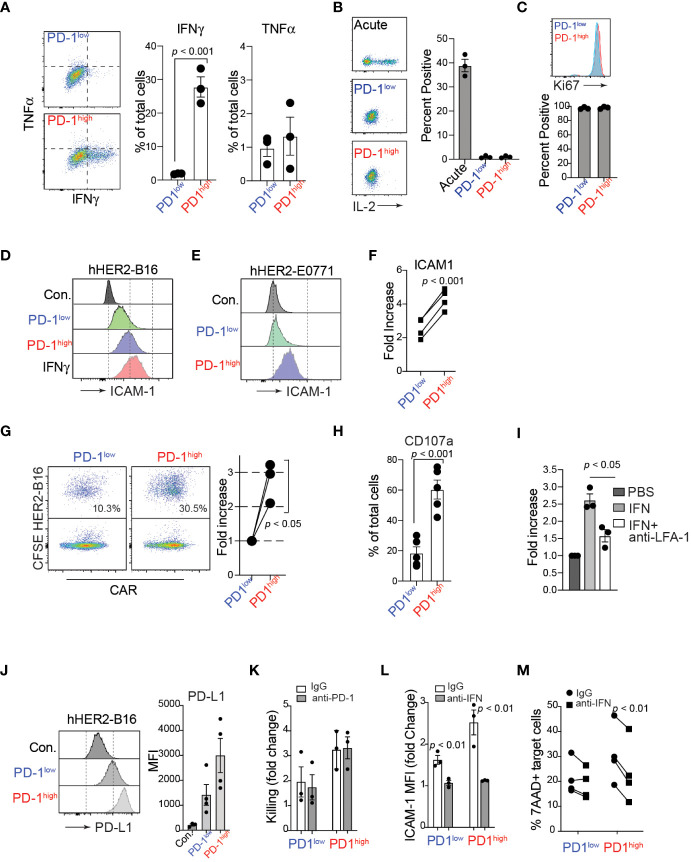
IFNγ production of PD-1^high^ CAR-T cells and enhanced engagement with target cancer cells. **(A)** Flow cytometry analysis of TNFα and IFNγ intracellular levels in PD-1^high^ and PD-1^low^ CAR-T cells after 4-hour co-cultures with target cancer cells in the presence of monensin (1:1000) and brefeldin A (1:1000). A representative experiment is shown from 3 independent experiments. On the right, quantification of the percent of PD-1^high^ and PD-1^low^ CAR-T cells producing TNFα and IFNγ is shown from 3 independent experiments performed in triplicate. *P* value was determined by unpaired, two-sided Student’s *t*-test. **(B)** Representative flow cytometry analysis (*left*) and quantification (*right*) of intracellular IL-2 staining after stimulation with plate-bound anti-CD3/CD28 antibodies for acute, PD-1^low^ and PD-1^high^ CAR T cells from 3 experiments performed in triplicate. **(C)** Representative flow cytometry analysis (*upper*) and quantification (*lower*) of intracellular Ki67 staining for sorted PD-1^low^ and PD-1^high^ CAR T cells from 3 independent experiments performed in triplicate. **(D, E)** Flow cytometry histograms of surface ICAM-1 expression on hHER2-B16 **(D)** and hHER2-E0771 **(E)** after 16 hour co-culture with PD-1^high^ and PD-1^low^ CAR-T cells, or with 100 ng/mL recombinant mouse IFNγ. **(F)** Quantification of the increase in hHER2-B16 ICAM-1 expression after co-culture with PD-1^high^ and PD-1^low^ CAR-T cells relative to untreated hHER2-B16 from 4 independent experiments in triplicate is shown. *P* value was determined by unpaired, two-sided Student’s *t*-test. **(G)** CAR-T cells were sorted on PD-1 expression and labeled with CellTrace FarRed and mixed with CFSE-labelled hHER2-B16 for 2 hours. Events double-positive for CellTrace FarRed and CFSE were measured using flow cytometry to assess the percent of CAR-T cells in conjugation with target hHER2-B16 (*left*). Quantification of the relative increase from PD-1^low^ to PD-1^high^ is shown on the right from 3 independent experiments. *P* value was determined by unpaired, two-sided Student’s *t*-test. **(H)** PD-1^high^ and PD-1^low^ CAR-T cells were cultured with CFSE-labelled target cancer cells for 4 hours in the presence of monensin (1:1000). The percent of cells positive for CD107a staining was measured for PD-1^high^ and PD-1^low^ CAR-T cells by flow cytometry from 5 independent experiments performed in triplicate. *P* value was determined by unpaired, two-sided Student’s *t*-test. **(I)** hHER2-B16 cells were treated with 100 ng/mL recombinant mouse IFNγ or PBS overnight. Cancer cells (CFSE) were used for the conjugation assay with CAR-T cells (CellTrace FarRed) in the presence or absence of anti-LFA-1 antibody. The fold increase in conjugation formation from PBS alone was quantified from 3 independent experiments. *P* value was determined by unpaired, two-sided Student’s *t*-test. **(J)** Representative flow cytometry histogram (*left*) and mean fluorescence intensity quantification (*right*) of surface PD-L1 expression on hHER2-B16 after coculture with PD-1^low^ or PD-1^high^ CAR T cells from 4 independent experiments performed in triplicate. **(K)** Quantification of target tumor cell killing relative to control T cells for PD-1^low^ and PD-1^high^ CAR T cells in the presence of 20 μg/mL anti-PD-1 antibody from 3 independent experiments performed in triplicate. **(L)** Quantification of the fold change in ICAM-1 MFI on target tumor cells after coculture with PD-1^low^ or PD-1^high^ CAR T cells with 20 μg/mL anti-IFNγ antibody from 3 independent experiments performed in triplicate. **(M)** Quantification of percent 7AAD+ target cancer cells after after coculture with PD-1^low^ or PD-1^high^ CAR T cells with 20 μg/mL anti-IFNγ antibody from 4 independent experiments performed in triplicate.

In addition to active cell migration to form stable conjugations with target cells, CAR-T cells can produce various cytokines, such as TNFα and IFNγ, in the tumor microenvironment. Importantly, recent studies have demonstrated that the adhesion between CAR-T cells and target cancer cells is directly regulated by IFNγ, which induces an upregulation of ICAM-1 on target tumor cells, promoting the formation of conjugations with CAR-T cells (44, 45). Consistent with these studies, after co-culturing with hHER2-expressing target cancer cells, we identified that a significantly higher proportion of PD-1^high^ hHER2-CAR-T cells than PD-1^low^ CAR-T cells produced IFNγ (**Figure 4A**). We also tested the ability of PD-1^high^ and PD-1^low^ CAR T cells to produce IL-2 following stimulation with plate-bound anti-CD3/CD28. Despite their lack of IL-2 production relative to acute *in vitro* activation, both PD-1^high^ and PD-1^low^ CAR T cells remained highly proliferative as measured by Ki67 staining (**Figures 4B, C**). Note that Ki67 is also expressed on recently divided and terminally exhausted T cell populations with decreased effector functions (41, 46). Furthermore, co-culture of PD-1^high^ hHER2-CAR-T cells with hHER2-B16 or hHER2-E0771 upregulated ICAM-1 expression on the target tumor cells to a level similar to that on B16 cells treated with recombinant IFNγ alone (**Figures 4D-F**). Enhanced ICAM-1 expression on target tumor cells led to an increase in conjugation formation with PD-1^high^ hHER2-CAR-T cells (**Figure 4G**) followed by greater lytic granule exocytosis (measured as CD107a expression), compared to PD-1^low^ CAR-T cells (**Figure 4H** and **Supplementary Figure 3**). IFNγ and ICAM-1 dependent conjugation formation between hHER2-CAR-T cells and target tumor cells was further confirmed by incubating tumor cells with recombinant IFNγ with/without an anti-LFA-1 blocking antibody (**Figure 4I**). Our data suggest that PD-1^high^ CAR-T cells are able to produce more IFNγ than PD-1^low^ CAR-T cells, resulting in robust upregulation of ICAM-1 on target cancer cells, which then supports more stable T cell-tumor cell conjugation formations to improve T cell-mediated killing. Consistent with ICAM-1 expression, coculture with PD-1^high^ and PD-1^low^ CAR-T cells induced PD-L1 expression on the target HER2-B16 cells (**Figure 4J**). Importantly, PD-1^high^ CAR-T cells maintain superior killing capacity despite the greater increase in PD-L1 expression on target cells (**Figures 4K**, **2A**). However, CAR-T cell-mediated cancer cell killing was not further enhanced with the addition of anti-PD-1 antibody (**Figure 4K**), indicating that PD-1/PD-L1 axis does not directly regulate CAR-T cell cytotoxicity *in vitro* (47). Instead, treatment of IFNγ neutralizing antibody during the coculture reduced ICAM-1 expression on target cancer cells and suppressed the killing function of CAR-T cells (**Figures 4L**, **4M**), indicating that CAR-T cell cytotoxicity is dependent on the production of IFNγ and subsequent ICAM-1 expression on their target cells (48). Importantly, enhanced IFNγ production and improved target cell killing by PD-1^high^ CAR T cells was observed in human anti-IL-13 receptor α2 (IL13Rα2) CAR-T cells co-incubated with a human melanoma cell (**Supplementary Figure 4**).

### Optimum cancer control by PD-1^high^ CAR-T cells is achieved with PD-1 blockade

**Figure 5:**
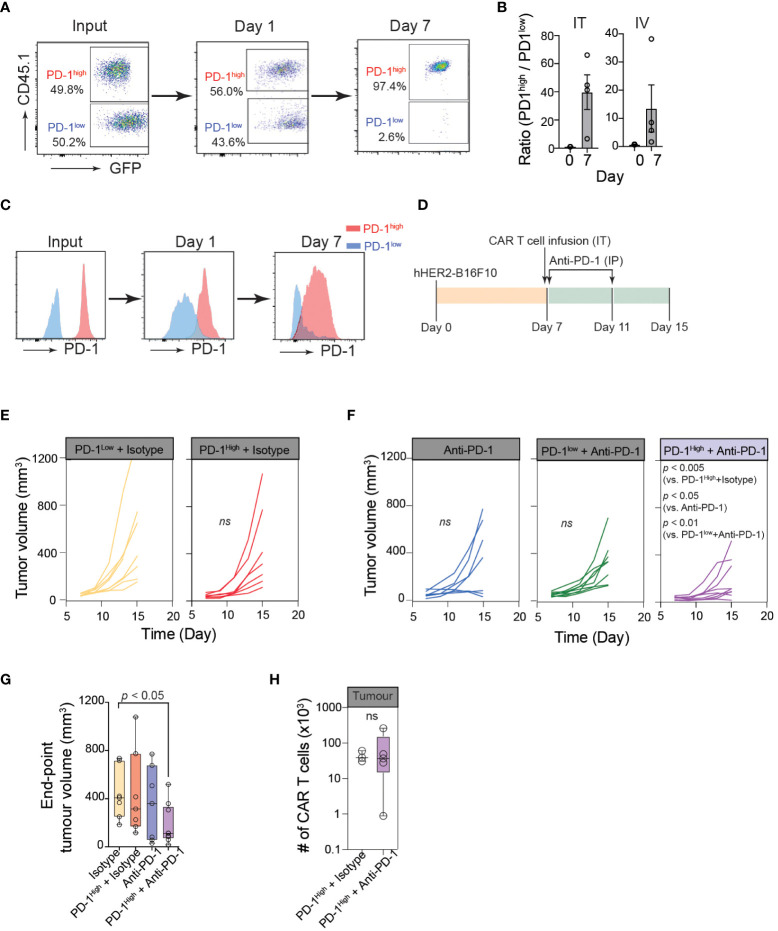
Optimum cancer control by PD-1^high^ CAR-T cells is achieved with PD-1 blockade. **(A)** Representative flow cytometric data showing PD-1^high^ (CD45.1^+^GFP^+^) to PD-1^low^ (GFP^+^) ratio at the time of injection (input), and the intratumoral PD-1^high^ to PD-1^low^ ratio 24 hours and 7 days post-intratumoral injection. **(B)** Quantification of PD-1^high^ to PD-1^low^ ratio at the time of injection (Day 0) and 7 days after both intratumoral injection (IT; left) or intravenous injection (IV; right) from 4 independent experiments. **(C)** Representative data of surface PD-1 expression for PD-1^high^ and PD-1^low^ CAR-T cells 1 day and 7 days post-intratumoral injection. **(D)** Schematic of experimental design for combination therapy. Mice were inoculated with hHER2-B16 on the mouse flank. PD-1^high^ or PD-1^low^ CAR-T cells were injected intratumorally on Day 7 and mice were given anti-PD-1 or isotype antibody injections I.P. on days 7 and 11. **(E**, **F)** Longitudinal tumor burden (volume, mm^3^) of hHER2-B16 tumor-bearing mice treated with 1x10^6^ PD-1^high^ or PD-1^low^ CAR-T cells **(E)** with or without a combination of 100 μg anti-PD-1 immune checkpoint blockade **(F)**. Tumor volume was calculated using the following formula: (tumor lx tumor w^2^x0.52), and tracked every 2 days. Isotype only *n*=8, anti-PD-1 only *n*=7, PD-1^high^ + isotype *n*=7, PD-1^high^ + anti-PD-1 *n*=9. *P* value was determined by ordinary One-Way ANOVA with a Tukey’s multiple-comparison post-test. **(G)** Quantification of tumor volume 8 days after PD-1^high^ CAR-T cell injection plus isotype or anti-PD-1 blockade, as well as isotype and anti-PD-1 alone. *P* value was determined by two-way ANOVA. **(H)** Quantification of intratumoral CAR-T cells per tumor for mice treated with PD-1^high^ CAR-T cells treated with Isotype (*n*=3) or anti-PD-1 antibodies (*n*=4). *P* value was determined by unpaired, two-sided Student’s *t*-test.

To further confirm the superior anti-cancer functions of PD-1^high^ CAR-T cells *in vivo*, we first performed a competitive tumor retention assay. For *in vivo* assays, we used human-HER2 (hHER2) transgenic (tg) C57BL/6 mice that overexpress the human erbB-2 gene under the mouse mammary tumor virus promoter (obtained from Genentech (32, 49)). hHER2 Tg mice bearing hHER2-B16 tumors received equal numbers (1:1) of PD-1^high^ hHER2-CAR-T cells (GFP^+^CD45.1^+^) and PD-1^low^ hHER2-CAR-T cells (GFP^+^CD45.2^+^) injected intratumorally (**Figure 5A**). The ratio of PD-1^high^/PD-1^low^ CAR-T cells remained close to 1 after 24 hours, but by day 7, the majority of intratumoral CAR-T cells were PD-1^high^ hHER2-CAR-T cells (**Figure 5A**). A similar change in the PD-1^high^/PD-1^low^ CAR-T cell ratio on day 7 was also observed at the tumor site after i.v. injection of hHER2-CAR-T cells (**Figure 5B**). More importantly, PD-1^high^ hHER2-CAR-T cells at the tumor site on day 7 did not exhibit diminished cell surface expression of PD-1 (**Figure 5C**). These results demonstrated a greater capability of PD-1^high^ CAR-T cells to migrate and persist at target tumor sites *in vivo*.

Because PD-1^high^ CAR-T cells outcompeted PD-1^low^ CAR-T cells in multiple anti-cancer functional assays, we speculated that PD-1^high^ CAR-T cells would show superior cancer control *in vivo*. However, in contrast to our prediction, hHER2 tumor-bearing mice adoptively transferred with PD-1^high^ hHER2-CAR-T cells did not show an improved tumor regression pattern compared to mice received PD-1^low^ hHER2-CAR-T cell infusion (**Figures 5D, E**). Similar to various other tumor cell lines, B16 cells dramatically upregulate PD-L1 expression *in vivo* (50). Therefore, it is possible that PD-1^high^ hHER2-CAR-T cells are more susceptible to PD-L1-mediated immune inhibition within the tumor microenvironment. Indeed, upon combination with an anti-PD-1 blocking antibody, tumor growth was significantly diminished in hHER2 tumor-bearing mice infused with PD-1^high^ hHER2-CAR-T cells, but not with PD-1^low^ hHER2-CAR-T cells (**Figures 5D, F, G**). Interestingly, PD-1 blockade did not lead to a significant increase in the PD-1^high^ hHER2-CAR-T cell number at the tumor site (**Figure 5H**), suggesting that the anti-PD-1 blocking antibody augmented the cytotoxic capability of PD-1^high^ hHER2-CAR-T cells *in vivo* but not the recruitment and/or retention of the T cells at the tumor site.

## Discussion

Our functional analysis of *in vitro* manufactured CAR-T cells based on their PD-1 expression revealed that the highest PD-1 expressers had enhanced cytotoxic function, with improved abilities to bind target cancer cells and release lytic granules. Despite their subdued migration pattern *in vitro*, PD-1^high^ CAR-T cells exhibited greater accumulation and retention in melanoma tumors than their PD-1^low^ counterparts *in vivo* and were more responsive to combination therapy with PD-1-targeting immune checkpoint blockade, helping to drive a potent early anti-tumor response.

In our CAR T cell activation and expansion model, both PD-1^high^ and PD-1^low^ CAR T cells were largely LY108- (a surrogate for TCF1), thus unlikely to be members of the progenitor exhausted T cell population (**Figures 1D, E**). They also displayed low surface expression of markers for previously identified “transitory” (CX_3_CR1+) and “dysfunctional” (CD101+) exhausted states that arise during chronic LCMV infection (51), suggesting that CAR-T cells do not fall into known exhausted subsets, despite heightened surface expression of inhibitory receptors such as PD-1, TIM3 and LAG3. Our functional analysis of PD-1^high^ cells shows that these cells produce low levels of IL-2 and TNFα, and remain highly proliferative, highly cytolytic, produce IFNγ, and persist within tumors, suggesting that PD-1^high^ CAR-T cells may be in the early stages of T cell dysfunction, but not fully exhausted (10).

Compared to PD-1^low^ CAR T cells, PD-1^high^ CAR T cells displayed elevated levels of lymph node homing adhesion molecule CD62L, often associated with central memory T cells. They also expressed slightly elevated levels of CXCR5, a chemokine receptor associated with distinct functional capabilities in a variety of disease contexts. Interestingly, superior cell survival and effector functions were reported in CXCR5+ T cells from follicular lymphoma patients (52). PD-1^high^ CAR T cells also expressed elevated levels of the IL-7 receptor, CD127, which is a marker of memory precursor effector cells (MPECs) that can give rise to long-lived memory cells (53, 54). Additionally, our live imaging of T cell - cancer cell interaction (Video 2) strongly suggests that the increased synapse stability is a feature of the dampened motility in PD-1^high^ CAR T cells. In addition to supporting the enhanced killing of target cells, the increased synapse duration in PD-1^high^ CAR T cells may provide extended CAR stimulation, and increase cell survival, as seen in antigen-specific CD8 T cells that become dependent on TCR stimulation (55). Together, all of these are features that may lend to the enhanced survival and persistence of PD-1^high^ CAR T cells shown in our *in vivo* studies. PD-1^high^ CAR T cells, despite continuous anti-CD3 stimulation and expression of multiple inhibitory receptors, seemingly fall more into the category of traditional memory subsets, or potentially long-lived effector cells, rather than the exhausted lineage.

Memory phenotypes and T cell stemness have been linked with the response in both CD19 CAR-T cell and solid tumor therapies, as this population has been linked with better engraftment in patients and responses against solid tumors (56). However, even if a stem-like phenotype may be optimal for early T cell proliferation and reactivation *in vivo*, it often cannot be attained in patients with certain cancer types who have been heavily pretreated in the clinic (57). Recent studies suggested important differences in the killing mechanisms of CAR-T cells between solid and liquid cancers; specifically, the IFNγR pathway is critical for solid tumor killing but dispensable for the killing of B-cell cancers (45). It is possible that elimination of solid tumors may require T cells with a unique phenotype that are more highly activated, produces more inflammatory cytokines such as IFNγ and are more efficiently recruited to tumor beds. Interestingly, a recent study using TCR transgenic therapy for pancreatic tumors showed that a responding patient had higher proportions of cells expressing the inhibitory receptors PD-1 and TIM3 and tissue-resident markers than the patient who failed to respond (58), and this cell phenotype has also been strongly linked with early response to immune checkpoint blockades (59). Thus, it is possible that modifying the manufacturing protocol to promote the highly activated CAR-T cell phenotype may be better going forward for solid tumors with immune checkpoint combination therapies depending on the type of tumor being targeted.

Exhausted CAR-T cells have shown anti-tumor functions *in vitro* but ultimately failed *in vivo* (60). Many clinical studies highlight that patients given infusion products showing features of T cell exhaustion are overall less likely to respond to T cell immunotherapy (4, 5). Consistent with these results, although our *in vitro* data showed that PD-1^high^ CAR-T cells outcompeted PD-1^low^ CAR-T cells in multiple anti-cancer functional assays, PD-1^high^ CAR-T cells did not show superior cancer control *in vivo* (**Figure 5**). Instead, our data suggest that patients with higher expression levels of inhibitory receptors (particularly PD-1) in their CAR-T cell infusion products are more susceptible to immune inhibition within the tumor microenvironment, thus would be strong candidates for combination therapy with checkpoint inhibitions to drive a stronger overall clinical response (5). In addition, most 2^nd^ generation CAR constructs with CD28 cytoplasmic domain may have the potential to tonically signal and become further exhausted, albeit to various degrees, accompanied by loss of effector functions (61, 62). In contrast, our study used the 3^rd^ generation HER2-CAR construct containing 4-1BB, suggesting this CAR construct does not induce strong tonic signals, thus not driving greater exhaustion programs (62).

The optimal regimens for both immune checkpoint blockade and solid tumor CAR-T therapy are still under active investigation, so the best way to combine these therapies is not yet well understood. Successful clinical response to immune checkpoint blockade has been linked with multiple features, such as IFNγ signatures, PD-1/PD-L1 expression, size and quantity of tumor-associated tertiary lymphoid organs, presence of B cells, and memory T cell phenotypes (59, 63–65). This immune information in individual patients would help define a model where checkpoint blockade may work best when a limited, yet ongoing immune response is already taking place. Based on our results, it is tempting to speculate that the combination of immune checkpoint blockades with a more highly activated CAR-T cell product extends the functionality of the highly activated CAR-T cells and potentially mitigates the negative functions of inhibitory receptors.

## Data availability statement

The raw data supporting the conclusions of this article will be made available by the authors, without undue reservation.

## Ethics statement

The studies involving human participants were reviewed and approved by University of Rochester Medical Center Institutional Review Board. The patients/participants provided their written informed consent to participate in this study. The animal study was reviewed and approved by University Committee on Animal Resources at the University of Rochester.

## Author contributions

CS and MK designed research. CS, YH, AD, AR, SM performed research. SG provided mouse protocols. PR provided CAR-T cell patient samples. CS and MK analyzed the data and wrote the paper. All authors contributed to the article and approved the submitted version.
