# Supplementary material for: PD-1Hi CAR-T cells provide superior protection against solid tumors
Source: Front Immunol. 2023 Jun 14;14:1187850. doi: 10.3389/fimmu.2023.1187850 (PMC10303811; doi:10.3389/fimmu.2023.1187850)
Supplement: Supplementary file 1 [file DataSheet_1.docx]

**
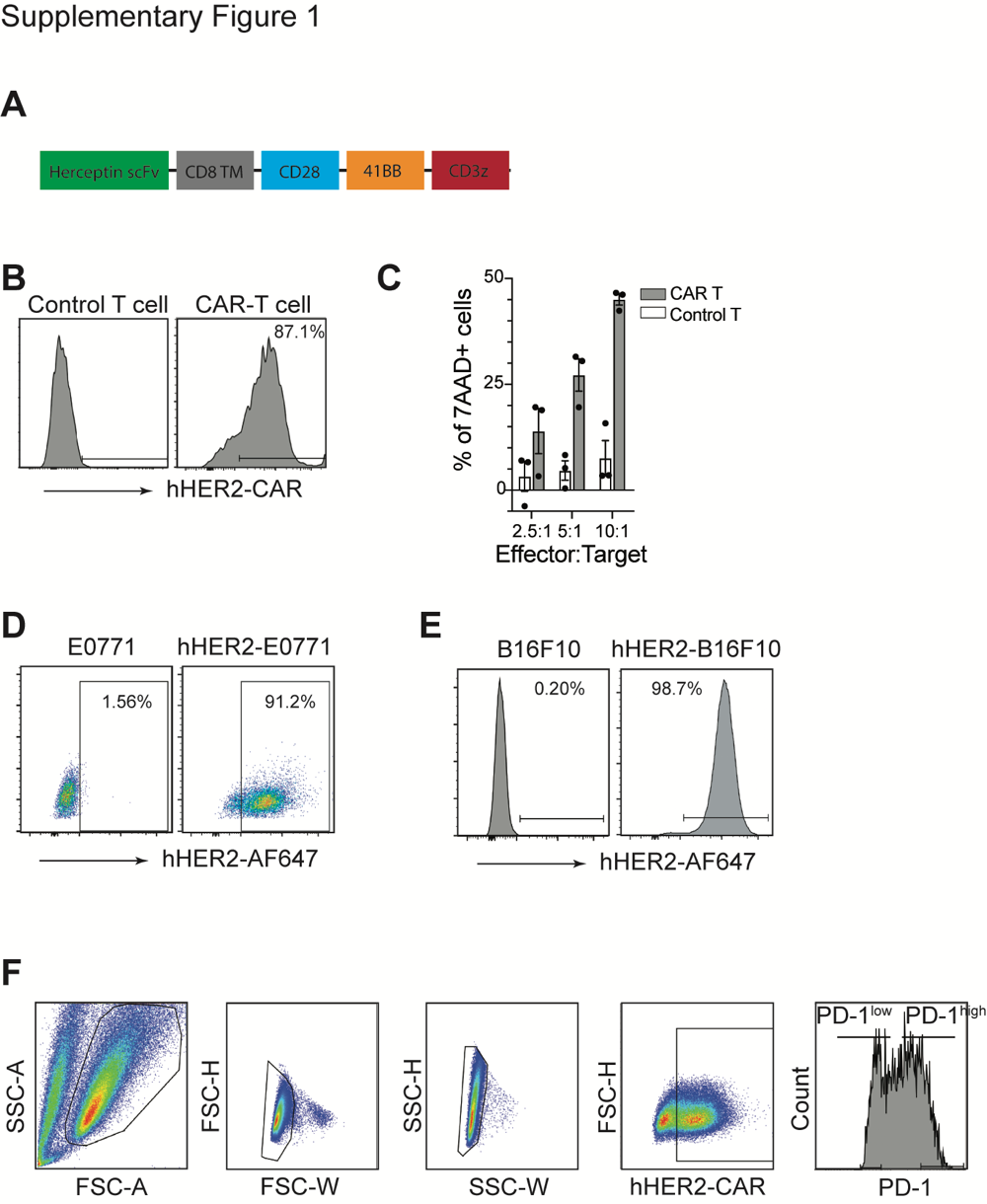
**

**Supplemental Figure 1**

HER2-CAR expression, cancer cell engineered HER2 expression, and sorting schema. **(A)** Schematic of the HER2-CAR construct. **(B)** Mouse CD8 T cells were activated and transduced with HER2-CAR retrovirus. CAR expression was detected after 2 μg Fc blocker (BD Biosciences) for 10 min at 4°C and incubated with recombinant human ErbB2/Her2 Fc chimera protein (R&D systems) at 4℃ for 30 min. After washing in PBS, the cells were incubated with PE-conjugated anti-human IgG Fc secondary antibody (eBioscience). **(C)** CAR T cells and non-transduced T cells were co-cultured with CFSE-labeled hHER2-E0771 at various effector to target ratios overnight. CFSE-positive target cells were collected and stained with 7-AAD to detect dead cells and assessed via flow cytometry. **(D, E)** Representative flow cytometry data showing surface human HER2 expression for hHER2-E0771 **(D)** and hHER2-B16F10 **(E)** stable cell lines generated as described in method section. **(F)** Gating strategy for PD-1^high^ and PD-1^Low^ CAR T cell sorting. Lymphocyte gate was drawn via FSC-A and SSC-A, followed by doublet discrimination. CAR-positive cells were gated from the single cell population, and then sorted upon the top and bottom 20% of PD-1 expression.

**
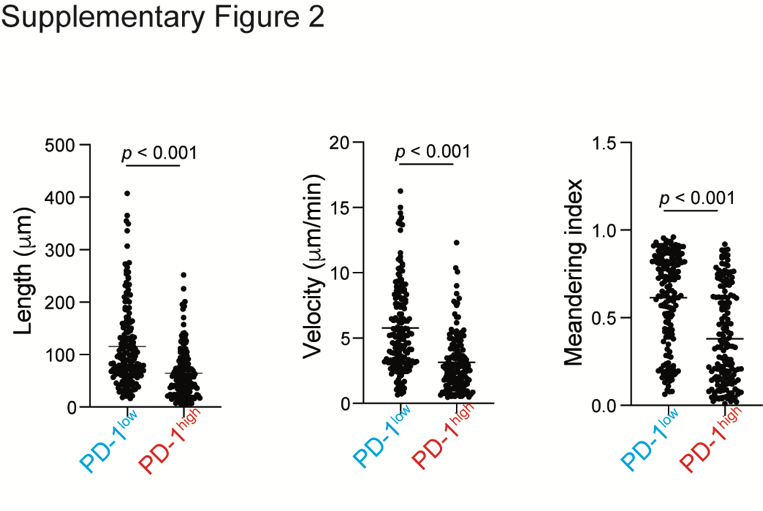
**

**Supplemental Figure 2**

Track length, velocity, and meandering index were calculated from PD-1^high^ and PD-1^low^ CAR-T cells migration on ICAM-1 and CXCL10 coated cover-glass. Solid black line indicates the mean, and *P* value was determined by unpaired, two-sided Student’s *t*-test.

**
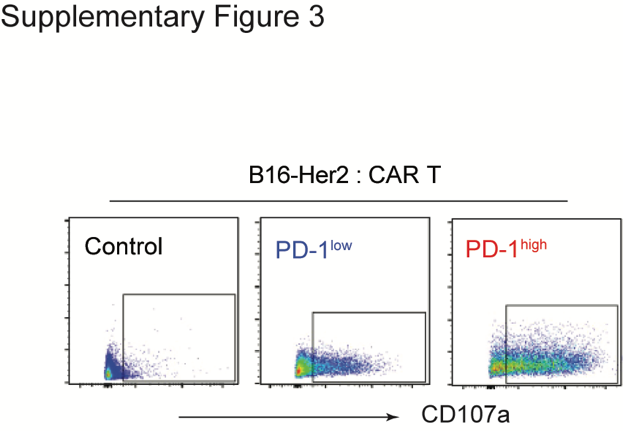
**

**Supplemental Figure 3**

Degranulation of PD-1^high^ and PD-1^Low^ CAR T cells. Representative flow cytometry data of surface CD107a expression after co-culture with target cancer cells. Sorted of PD-1^high^ and PD-1^low^ CAR T cells and non-transduced T cells were co-cultured with CFSE-stained hHER2-B16 for 4 hours in the presence of Monensin (1:1000) and PECy7-conjugated anti-CD107a (1 uL per mL of media). Cell surface expression of CD107a was assessed by flow cytometry.

**
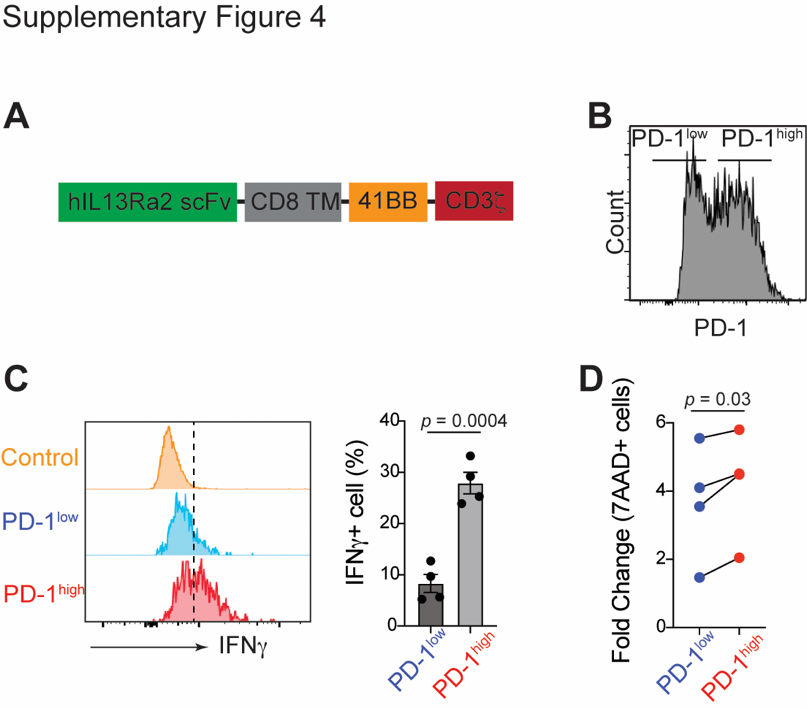
**

**Supplemental Figure 4**

Human IL13Rα2 CAR. **(A)** Schematic of the IL13Rα2 CAR construct. **(B)** Representative flow cytometry data showing PD-1 expression after CAR T cell activation. **(C)** Representative flow cytometry data (*left*) and quantification (*right*) of the percent human CAR T cells expressing intracellular IFNγ. **(D)** Killing of A375 melanoma cells (7-AAD+) by human PD-1^high^ and PD-1^low^  IL13Rα2 CAR T cells. Solid black line indicates the mean, and *P* value was determined by both unpaired (C) and paired (D) two-sided Student’s *t*-test.

**Supplemental Movie 1**

Representative movie of PD-1^Low^ and PD-1^High^ HER2-CAR T cells migrating on ICAM-1 and CXCL12.

**Supplemental Movie 2**

PD-1^Low^ (blue) and PD-1^High^ (red) HER2-CAR T cells migrating on ICAM-1 and CXCL12 with hHER2-B16 cancer cells (green).

**Supplemental Movie 3**

PD-1^Low^ (red) and PD-1^High^ (green) HER2-CAR T cells migrating in 50% Matrigel with HER2+ BT474 tumor spheroid.
